# Large-scale Synthesis of β-SiC Nanochains and Their Raman/Photoluminescence Properties

**DOI:** 10.1007/s11671-010-9787-7

**Published:** 2010-09-26

**Authors:** Alan Meng, Meng Zhang, Weidong Gao, Shibin Sun, Zhenjiang Li

**Affiliations:** 1 Key Laboratory of Eco-chemical Engineering, Ministry of Education; College of Chemistry and Molecular Engineering, Qingdao University of Science and Technology, 266042 Qingdao, People's Republic of China; 2 College of Electromechanical Engineering, Qingdao University of Science and Technology, 266061 Qingdao, People's Republic of China

**Keywords:** Nanochain, Chemical vapor reaction, Growth mechanism, Optical property

## Abstract

Although the SiC/SiO_2_ nanochain heterojunction has been synthesized, the chained homogeneous nanostructure of SiC has not been reported before. Herein, the novel β-SiC nanochains are synthesized assisted by the AAO template. The characterized results demonstrate that the nanostructures are constructed by spheres of 25–30 nm and conjoint wires of 15–20 nm in diameters. Raman and photoluminescence measurements are used to explore the unique optical properties. A speed-alternating vapor–solid (SA-VS) growth mechanism is proposed to interpret the formation of this typical nanochains. The achieved nanochains enrich the species of one-dimensional (1D) nanostructures and may hold great potential applications in nanotechnology.

## Introduction

Controlled, rational, and designed growth of 1D nanostructures, such as nanowire, nanorod, nanotube, and nanobelt have attracted considerable attentions in nanotechnology due to the distinct potential applications in functional electronic, photonic, and mechanical nanodevices [1-4].

1D SiC nanostructures, as an outstanding wide-gap semiconducting materials [5-8], have exhibited great applications in composite materials, optical circuits, light-emitting diodes, field-emission devices, and hydrogen storage [9-12]. **These properties make SiC a promising candidate for various applications in nanoscale photoelectronic device **[13]. As known to all, some intrinsic properties are directly connected with the specific morphologies, much effort has being devoted to the synthesis and applications of various 1D SiC nanostructures, and different types of SiC nanostructures have been synthesized and reported in present literatures [14-23]. Incentived by the development of novel 1D SiC nanostructure to pursuit its unique property, a simple **chemical vapor reaction (CVR)** approach combined with nanoporous AAO template for SiC crystal growth control was successfully developed. **Herein, the SiC nanostructures were obtained via the chemical vapor process between the C (vapor) pyrolyzed from the C**_**3**_**H**_**6**_**and SiO (vapor) generated by a solid–solid reaction between milled Si-SiO**_**2**_**; therefore, the process was called after chemical Vapor Reaction (CVR).** The CVR approach has been well revealed for the synthesis of SiC nanostructures due to the inherent advantages of simple process, low cost, lower temperature, catalyst free and high purity quotient [24].

Just recently, we have successfully synthesized periodic composite SiC/SiO_2_ beaded nanostructures (SiC nanowires and SiO_2_ nanospheres) on carbon substrate via the CVR approach [25]. Wei et al. [26]. have synthesized 1D SiC/SiO_2_ nanochain heterojunctions are composed of 3C-SiC strings and SiO_2_ beads via microwave method; however, the chained homogeneous nanostructure of single-crystalline SiC has not been reported erenow. In this paper, the nanostructures of the product are constructed by spheres of 25–30 nm and conjoint wires of 15–20 nm in diameters. [111] is the typically preferred growth direction with high density of stacking faults. Moreover, the as-gained nanostructures may have great application in improving the properties of the nano-composite materials due to the strong adhesion between the contoured surface nanowires and matrix comparing to the smooth-surface nanowires [27]. Synthesis, characterizations, the corresponding Raman spectroscopy and photoluminescence measurement are also reported and discussed in this paper. A SA-VS growth mechanism is further proposed to interpret the growth mechanism of the uniform single-crystalline SiC nanochains.

## Experimental

The nanoporous AAO template was prepared by a two-step aluminum anodic oxidation process. Prior to anodizing, the high-purity aluminum thin sheets were annealed at 450°C for 2 h, rinsed in distilled water, then electro-polished to achieve a smooth surface. Subsequently, the samples were anodized in 0.3 M oxalic acid (40 V, 17°C, 4–5 h, Al sheet as an anode). First, the anodized layer was removed by etching in a mixture of phosphoric acid and chromic acid at 60°C for 8–10 h. Second, the samples rinsed in distilled water and oxalic acid was anodized again in 0.3 M oxalic acid (40 V, 16°C, 7–9 h, Al sheet as an anode). After the anodizing, the unwanted aluminum matrix was dissolved in HgCl_2_ solution at room temperature. Finally, the template was rinsed with distilled water and immersed in 5% phosphoric acid for about 20–40 min at 32°C to adjust the pore diameter and remove the barrier layer at the bottom of nanoholes.

Typical CVR processes were carried out as follows: First, the AAO template was put at the bottom of the homemade reaction chamber and supported by a piece of carbon cloth. **The mixture of Si and SiO**_**2**_**powder (molar ration 1:1) was placed over the AAO template, and a piece of carbon cloth was inserted between the template and the mixture powder. Another piece of carbon cloth was situated over the powder.**

Then the chamber was placed into a vertical furnace, before heating, the furnace was purged 2–3 times with high-purity argon (Ar) using a rotary vacuum pump, the temperature of furnace was increased to 1,230°C from room temperature at a mean speed of 450–500°C.h^-1^ held the top temperature for 10-15 min. Meanwhile, the steady flow of C_3_H_6_ at 0.1–0.2 sccm from the bottom of the graphite reaction chamber was started and maintained it for 5–10 min. Finally, the power was switched off and the furnace was cooled to room temperature.

The synthesized AAO template and SiC nanochains were characterized by a JEOL JSM-6 FESEM equipped with elemental EDX equipment. Further detailed structural information was obtained by a JEOL-2010 HRTEM and SAED. XRD pattern was recorded by a Rigaku D/max-2,400 X-ray diffractometer. Raman spectra were measured by a Renishaw 2,000 micro-Raman spectrometer. Photoluminescence spectra were performed in a Hitachi F-4500 fluorescence spectrophotometer.

## Results and Discussion

**Figure 1:**
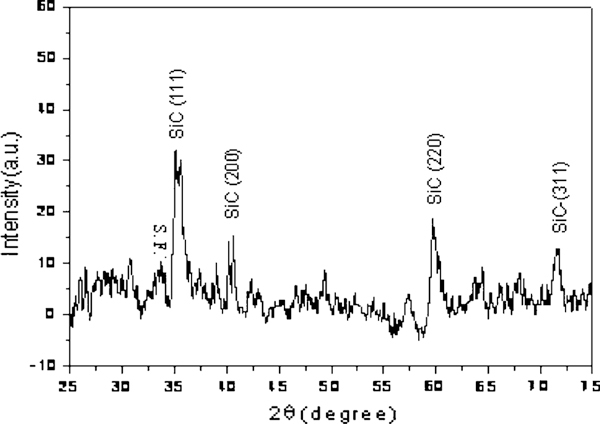
**Representative XRD pattern of the products grown on the AAO template indicating the crystalline β-SiC**.

The representative XRD pattern of the products grown on AAO template is also observed in Figure 1, suggesting that β-SiC is the only crystalline phase. The major diffraction peaks could be assigned to the (111), (200), (220), and (311). These values are in well agreement with the known values for β-SiC (JCPDS Card. No.29-1129). The low-density peak is due to the crystal stacking faults [28]. Moreover; the amorphous background in the pattern is caused by the AAO template.

**Figure 2:**
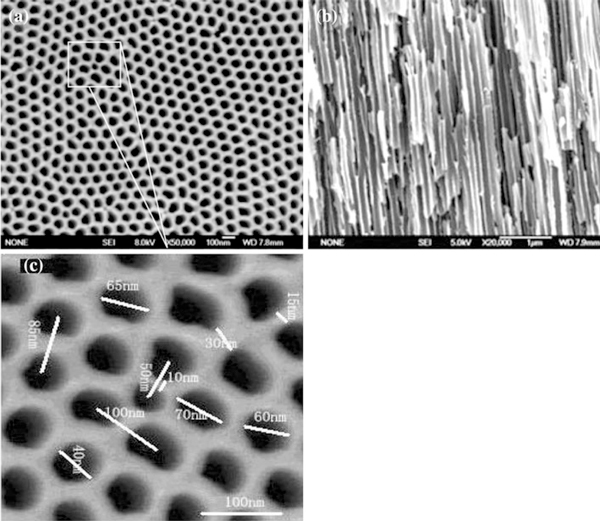
**Typical FESEM images of the ordered nanopores AAO template prepared by aluminum anodic oxidation processes**. **a** The top surface image. **b** View of the vertical section. **c** The enlarged image of marked area in Figure 2a.

Typical FESEM image scanned from the top surface of the AAO template is distinctly shown in Figure 2a. The well-ordered pores are clearly observed for the prepared template. Figure 2b is the side view of the vertically cut template, which clearly shows the AAO template possessing coterminous nanopores parallel to each other and perpendicular to the surface of the template. Figure 2c is the enlarged image of rectangular area in Figure 2a, it can be observed that AAO nanopores are inhomogeneously distributed with diameters of 30–70 nm and distances between the neighboring pore of 10–30 nm.

**Figure 3:**
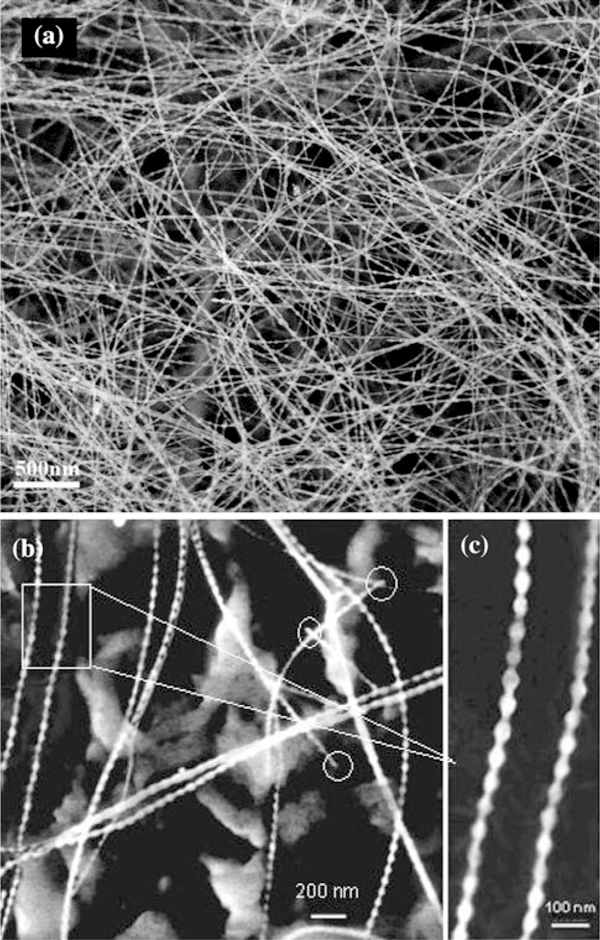
**Representative low-magnification (a) and high-magnification (b, c) FESEM images of the nanochains grown on AAO template via CVR approach**.

**Figure 4:**
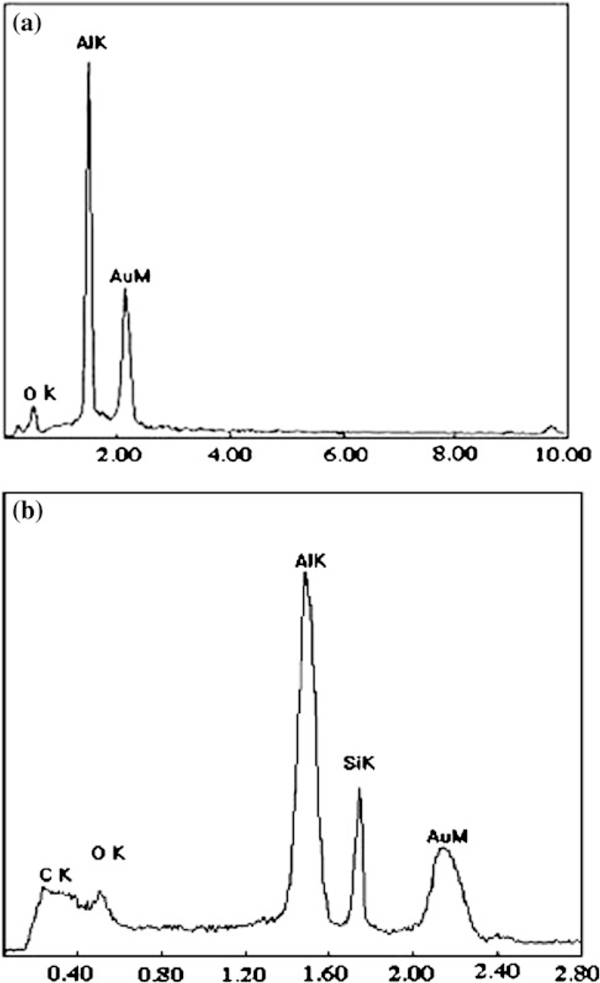
**EDX spectrums of the AAO template (a) and SiC nanochains (b)**.

Low-magnification FESEM image (Figure 3a) shows the nanochains grown on the AAO template via CVR approach, which are quite different from the traditional smooth nanowire. High-magnification FESEM image further reveals that the nanostructures in Figure 3b and 3c were the perfect beaded morphology with sphere size of 25–30 nm evenly and connected by the wire of 15–20 nm in diameter. No catalyst particles are attached onto the tips of the as-grown nanochains that are marked by the white circles, so we generally believe the growth process is induced by VS mechanism. In addition, the EDX spectrums (Figure 4) reveal that the AAO template is composed of Al and O elements, the quantitative analysis indicates the atomic ratio of Al and O is approximately 2:3, corresponding to the stoichiometric composition of Al_2_O_3_. The nanochains are composed of Si and C, and the molecular ratio of Si/C calculated from the EDX data is about 1:1, corresponding to the stoichiometric composition of SiC. The appearance of Al and O peaks is due to the existence of AAO substrate.

**Figure 5:**
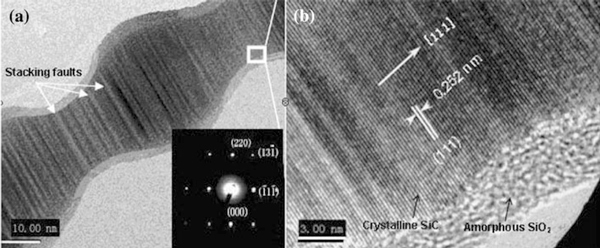
**a Low-magnification and b high-magnification HRTEM images of the SiC nanochains**. The inset image in (**a**) is the corresponding SAED patterns.

Furthermore, HRTEM image (Figure 5) provides the detailed structural information of the products. As shown in Figure 5a, the perfect SiC nanochain has spherical size of 25–30 nm and wire between adjacent spheres of 15–20 nm. Also, the stacking faults could be clearly observed within the nanostructure marked by arrows, suggesting that the SiC crystal possess a high density of defects. Additionally, during the cooling process, the residual oxygen and SiO vapors in the reaction chamber would also react to produce SiO_2_, which will then diffuse, precipitate, and nucleate easily on the surface of SiC nanostructure, otherwise, as the activity of the SiC nanostructures at the nanoscale, the surface of the products may be oxidized to be SiO_2_ in small amounts at room temperature after the reaction. Finally, both of above two reasons may result in the formation of very thin amorphous SiO_2_ layer, as shown in Figure 5b SiC nanostructures are actually wrapped by a very uniform thin SiO_2_ layer of 2–5 nm. Figure 5b distinctly depicts the spacing within the crystal between two adjacent lattice planes is 0.252 nm, indicating the crystal grows along [111] direction. These values are in well agreement with the known values for β-SiC crystal. The inset image in Figure 5a shows the corresponding SAED patterns, the sharp and regular single diffraction spots clearly indicate that the crystallized structure which could be indexed to the cubic crystalline β-SiC.

**Compared with the SiC nanowire arrays in our former work [**[24]**], we supposed that the position of the AAO template and the reaction time have palyed important roles in determining the morphology of the products. The nanoarrays can be synthesized just during short reaction time and the low reaction rate; moreover, the AAO template must be placed over the milled powders, and the detailed growth mechanism has been depicted in the relevant paper. The SiC/SiO**_**2**_**composite nanostructures [**[25]**] were synthesized without using any template or catalyst and two-stage vapor–solid growth mechanism was proposed. The first stage is the formation of the SiC nanowires within stacking faults, and the second stage is the formation of the SiO**_**2**_**spheres at the faults position. Moreover, the distribution of the gas over the carbon substrate is uniform, and the substrate could not limit the concentration of the reactant.**

Chen et al. have analyzed that the fluctuations of the growth conditions could influence the morphology of a nanostructure during the VS growth process [29]. The detailed depict has been reported in our other paper [30]. Typically, four possible reactions happened during the SiC nanostructure formation, as follows:

(1) $$\text{Si}+{\text{SiO}}_{2}\to 2\text{SiO}$$

(2) $${\text{C}}_{3}{\text{H}}_{6}\to 3\text{C}\left(\text{v}\right)+3{\text{H}}_{2}$$

(3) SiO+2C→SiC(s)+CO(v)

(4) $$\text{SiO}+3\text{CO}\to \text{SiC}\left(\text{s}\right)+2{\text{CO}}_{2}$$

**Figure 6:**
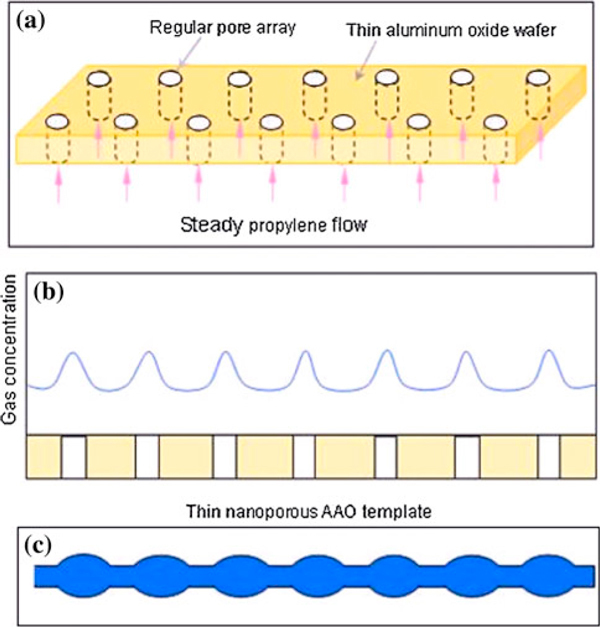
**a Schematic illustration for the bottom-up C3H6 flowing through the ordered nanoporous AAO template**. **b** The simulative carbon concentration curve upon the AAO template during the synthesis process. **c** The stimulant SiC nanochains.

Herein, **in order to achieve longer reaction time and higher reaction, the AAO template was placed at the bottom of the reaction chamber to prepare the SiC nanochains;** we believe that the nanoporous AAO template has played a key role in the synthesis of SiC crystal. In order to interpret the formation process, a reasonable SA-VS growth mechanism is proposed and schematically shown in Figure 6. The original nucleate and growth of the SiC crystal could be induced by the micro-particles of Si, SiO_2_ and surface defects of AAO template via a traditional VS growth mechanism [31]. While with the C_3_H_6_ gas flow introduced into the chamber and encountered with the AAO template, the flow direction of C_3_H_6_ gas is guided by the nanopore channels (Figure 6a). Under the controlled flow speed of C_3_H_6_ gas, it is reasonable that the concentration of carbon decomposed from C_3_H_6_ gas form a regular and alternate gradient distribution over the whole template plane. A maximum carbon concentration may probably occur over the around center of the nanopores, as the schematic simulated alternative carbon concentration curve over the template shown in Figure 6b. Thus, during the further growth of SiC nanostructure along [111] direction, the growth of SiC crystal would be definitely affected by the change of carbon concentration, the maximum concentration is convinced to induce the maximum growth speed of SiC crystal, and finally the maximum diameter of formed SiC nanochains. Correspondingly, the thinner SiC nanostructures will be obtained following the lower carbon concentration. After continuous vs growth process, the diameter alternating SiC nanochains are obtained at last, as is schematically shown in Figure 6c. Owing to the nanoporous AAO template and the controlled gaseous flow of C_3_H_6_, the SiC nanochains could be formed by the SA-VS growth mechanism. In addition, the same experiment process was also performed by the use of carbon substrate with smooth surface instead of nanoporous AAO template, only smooth SiC nanowire while no nanochain was observed. This strongly proved that proposed SA-VS growth mechanism of SiC nanochain was assisted by the nanoporous AAO template.

**Figure 7:**
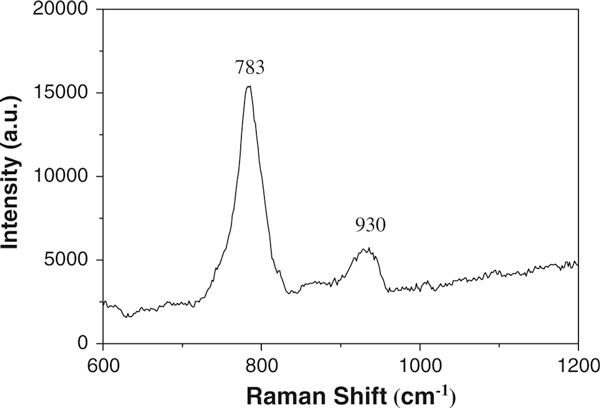
**Raman spectra of the SiC nanochains**.

A typical Raman spectrum of as-achieved SiC nanochains is shown in Figure 7. Two feature peaks at around 783 and 930 cm^-1^ could be assigned to the TO and LO phonon peaks of cubic SiC crystal, respectively, and a shift of 13 cm^-1^ and 42 cm^-1^ are observed comparing with the modes of bulk SiC [32,33]. When compared with that of SiC whisker, a shift of 11 cm^-1^ is observer for the TO peak [34]. The unique physical properties may be caused by the size confinement effect, inherent stacking faults, and novel chained morphology of SiC nanostructures [35]. Otherwise, slight alloying may be also the reasons to the peaks shift of the Raman spectrum.

**Figure 8:**
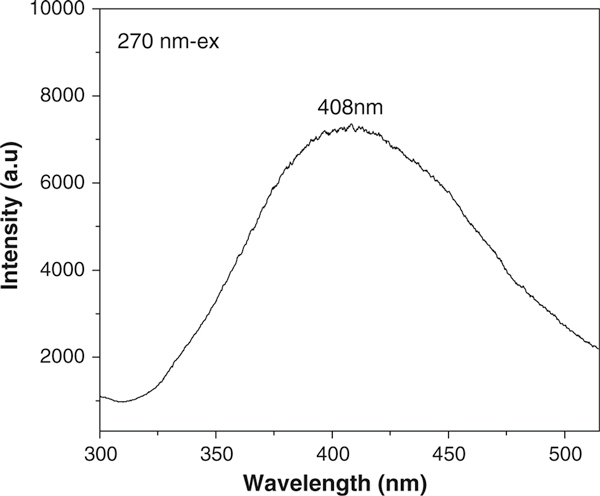
**Room-temperature photoluminescence spectrum of the SiC nanochains**.

Figure 8 displays a room-temperature PL spectrum of the as-obtained products. When excited with light from a xenon source (excitation wavelength of 270 nm), the typical nanostructures show emission band centered at 408 nm, which is in accordance with the value of 3C-SiC nanobelts [16] and SiC nanoneedles [36]. Compared with the previously reported luminescence from the aligned SiC NWs, the emission peak is obviously shifted [37]. Similar emission peaks at about 390 nm were also reported for the SiC/SiO_2_ nanochain heterojunctions, which was attributed to the neutral oxygen vacancy formed at the interface boundary of SiC/SiO_2 _[26]. In the present work, the SiC nanochains were uniformly wrapped by a thin SiO_2_ amorphous film. Hence, we proposed that the wide PL peak may be due to the following reasons: altering diameter of the nanostructures, stress at the SiC/SiO_2_ interface boundary, the size confinement effect and high density of defects including stacking faults within the SiC nanostructures.

## Conclusions

In summary, a simple CVR approach assisted by the nanoporous AAO template is successfully developed to form the single-crystalline β-SiC nanochains using Si-SiO_2_ powder and C_3_H_6_ gas as raw materials. The as-obtained nanostructures with spheres of 25–30 nm and conjunct wires of 15–20 nm in diameters typically possess novel chained homogeneous nanostructure which is different from the products have been reported. A SA-VS growth mechanism is proposed to interpret the formation of the SiC nanochain. This approach could also be applied to synthesize nanochains of the other materials. Raman and PL characterizations confirm the unique optical properties which mean the products may hold great application in functional nano-devices.
